# Involvement of aberrantly activated HOTAIR/EZH2/miR-193a feedback loop in progression of prostate cancer

**DOI:** 10.1186/s13046-017-0629-7

**Published:** 2017-11-15

**Authors:** Zhixin Ling, Xiaoyan Wang, Tao Tao, Lei Zhang, Han Guan, Zonghao You, Kai Lu, Guangyuan Zhang, Shuqiu Chen, Jianping Wu, Jinke Qian, Hui Liu, Bin Xu, Ming Chen

**Affiliations:** 1 Department of Urology, Affiliated Zhongda Hospital of Southeast University, Nanjing, Jiangsu 210009 China; 2 Surgical Research Center, Institute of Urology, Medical School of Southeast University, Nanjing, Jiangsu 210009 China; 3 Department of Nursing, Affiliated Zhongda Hospital of Southeast University, Nanjing, Jiangsu 210009 China; 4 Department of Urology, Anhui Provincial Hospital, Anhui Medical University, Hefei, 230001 China; 5 Department of Urology, the First Affiliated Hospital of Bengbu Medical College, Bengbu, 233004 China; 6 Department of Urology, Binhai People’s Hospital, Yancheng, Jiangsu 224500 China

**Keywords:** Prostate cancer, MiR-193a, HOTAIR, EZH2, Progression

## Abstract

**Background:**

Though androgen deprivation therapy is the standard treatment for prostate cancer (PCa), most patients would inevitably progress to castration-resistant prostate cancer (CRPC) which is the main cause of PCa death. Therefore, the identification of novel molecular mechanism regulating cancer progression and achievement of new insight into target therapy would be necessary for improving the benefits of PCa patients. This study aims to study the function and regulatory mechanism of HOTAIR/EZH2/miR-193a feedback loop in PCa progression.

**Methods:**

MSKCC and TCGA datasets were used to identify miR-193a expression profile in PCa. Cell Counting Kit-8 (CCK-8) assays, colony formation, invasion, migration, flow cytometry, a xenograft model and Gene Set Enrichment Analysis were used to detect and analyze the biological function of miR-193a. Then, we assessed the role of HOTAIR and EZH2 in regulation of miR-193a expression by using plasmid, lentivirus and small interfering RNA (siRNA). Luciferase reporter assays and chromatin immunoprecipitation assays were performed to detect the transcriptional activation of miR-193a by EZH2 and HOTAIR. Further, qRT-PCR and luciferase reporter assays were conducted to examine the regulatory role of miR-193a controlling the HOTAIR expression in PCa. Finally, the correlation between HOTAIR, EZH2 and miR-193a expression were analyzed using In situ hybridization and immunohistochemistry.

**Results:**

We found that miR-193a was significantly downregulated in metastatic PCa through mining MSKCC and TCGA datasets. In vitro studies revealed that miR-193a inhibited PCa cell growth, suppressed migration and invasion, and promoted apoptosis; in vivo results demonstrated that overexpression of miR-193a mediated by lentivirus dramatically reduced PCa xenograft tumor growth. Importantly, we found EZH2 coupled with HOTAIR to repress miR-193a expression through trimethylation of H3K27 at miR-193a promoter in PC3 and DU145 cells. Interestingly, further evidence illustrated that miR-193a directly targets HOTAIR showing as significantly reduced HOTAIR level in miR-193a overexpressed cells and tissues. The expression level of miR-193a was inversely associated with that of HOTAIR and EZH2 in PCa.

**Conclusion:**

This study firstly demonstrated that miR-193a acted as tumor suppressor in CRPC and the autoregulatory feedback loop of HOTAIR/EZH2/miR-193a served an important mechanism in PCa development. Targeting this aberrantly activated feedback loop may provide a potential therapeutic strategy.

**Electronic supplementary material:**

The online version of this article (doi: 10.1186/s13046-017-0629-7) contains supplementary material, which is available to authorized users.

## Background

Prostate cancer is the most commonly diagnosed malignancy in American men and is one of leading causes of cancer-related death among aging population [1]. The biggest challenge for PCa treatment is that most patients would inevitably progress to castration-resistant prostate cancer within 2 years’ androgen deprivation therapy, which is considered as main cause of prostate cancer patient death. A wide spectrum of genetic aberrations was associated with PCa development, however the exact molecular mechanism underlying PCa progression remains unclear.

Enhancer of zeste homolog 2 (EZH2) has been widely studied in the area of cancer epigenetics in recent years [2, 3]. EZH2 was first observed in cancer when it was identified as one of the most elevated genes in metastatic PCa and closely correlated with poor prognosis [4]. Given its dysregulation in PCa and its oncogenic role in PCa cells proliferation and metastasis, substantial efforts have been dedicated to identify its underlying molecular regulatory mechanisms and its potential therapy application in PCa. EZH2 is one of the core enzymatic subunit of histone methyltransferase polycomb repressor complex 2 (PRC2) which methylates lysine27 of histone H3 (H3K27) to promote transcriptional silencing of many tumor suppressive genes [5, 6]. In addition, several studies also demonstrated the PRC2-independent function of EZH2 in transcriptional activator rather than repression [7, 8]. A wide spectrum of genetic as well as epigenetic aberrations are associated with cancer development, many of which are associated with histone methyltransferase EZH2 dysregulation. However, the exact functional role of EZH2-mediated epigenetic silencing of tumor suppressive miRNAs in prostate cancer progression has not been systematically studied.

The action of microRNAs (miRNAs) has been studied in detail as destabilizers and repressors of translation of protein-coding transcripts (mRNAs) to regulate diverse biological function including proliferation, differentiation, apoptosis, metabolic progression in various cancers including PCa. MicroRNA-193a (miR-193a) was initially discovered in 2008, and it was identified as a tumor suppressive miRNA in oral carcinoma, lung cancer, colorectal cancer, and malignant pleural mesothelioma [9–12]. It has been reported that DNA methylation is associated with miR-193a downregulation in acute myeloid leukemia, hepatocellular carcinoma, non-small cell lung cancer, and oral cancer [9, 13–15]. In a previous miRNA microarray analysis, we detected a panel of miRNAs, including miR-193a, whose expression are downregulated in CRPC clinical samples [16]. However, the exact biological function of miR-193a in tumorigenesis of PCa remains largely unknown. Moreover, the molecular mechanism of miR-193a silencing in PCa is still unclear.

Long non-coding RNAs (lncRNAs) are defined as non-protein coding transcripts longer than 200 nucleotides. They were initially argued to be spurious transcriptional noise. However, increasing discoveries of dysregulated lncRNA expression among various cancer types implicated that aberrant lncRNA expression may be major contributor to tumorigenesis [17, 18]. For last decade, lncRNA HOX transcript antisense RNA (HOTAIR) has attracted considerable scientific attention in numerous cancers. Accumulating evidence indicate that HOTAIR may function as an oncogene in the malignant progression of various cancers including prostate cancer [19–22], indicating its potential role of effective therapeutic target in cancers. HOTAIR could directly binds to the AR protein to prevent its ubiquitination and protein degradation, thereby promoting PCa cell growth and invasion [22]. And HOTAIR is well known for its interacting with key epigenetic regulators such as PRC2 and histone demethylase LSD1 to induce gene silencing and chromatin dynamics, which appears to be misregulated in a variety of cancers [23, 24], whereas it’s regulatory role of genes expression in prostate cancer remains limited.

Furthermore, numerous studies during last decade have illustrated that lncRNAs can be targeted by miRNAs to reduce lncRNA stability. LncRNA MALAT1 can be targeted by microRNA-9 in the human primary glioblastoma cell line U87MG and the human Hodgkin cell line L428 [25]. Another cancer-related lncRNA PTCSC3 is targeted and repressed by miR-574-5p in thyroid cancer cell lines [26]. It is reported that recruitment of let-7 by HuR reduced the HOTAIR stability and decreased its expression [27]. So far, few studies have revealed the potential miRNAs that can directly negatively regulate HOTAIR expression in PCa.

In this study, we systemically investigated the biological functions of miR-193a and results suggested that miR-193a functions as a tumor suppressor in PCa. And HOTAIR could interact with EZH2 to repress miR-193a expression by epigenetic modification. On other hand, miR-193a directly targets HOTAIR and reduces HOTAIR expression in PCa. This study demonstrated that the autoregulatory feedback loop of HOTAIR/EZH2/miR-193a plays a key role in PCa development.

## Methods

### Cell culture and tissue collection

The human PCa cell line DU145 and PC3 were purchased from ATCC (Manassas, VA, USA). Cells were cultured in RPMI-1640 (Gibco, Grand Island, New York State, USA) supplemented with 10% fetal bovine serum (Gibco), 100 U/ml penicillin, and 100μg/ml streptomycin at 37 °C in a 5% CO_2_ incubator. PCa (*n* = 31) samples were collected from Zhongda Hospital Affiliated with Southeast University. PCa tissues were obtained from early-stage patients who underwent radical prostatectomy and never received previous treatment. Clinicopatholigical features of these specimens were examined by senior pathologist and diagnosed according to pathological evidence. All the patients whose samples enrolled in this study have been informed and signed consents for their tissues used in scientific research, and this research also obtained approval from ethics of Zhongda Hospital Affiliated with Southeast University.

### Oligonucleotides, plasmid, lentivirus, and cell transfection

Oligonucleotides were chemically synthesized by GenePharma (Shanghai, China) according to the sequences of has-miR-193a mimic (miR-193a), 5′-AACUGGCCUACAAAGUCCCAGU-3′ (sense), 5′-UGGGACUUUGUAGGCCAGUUUU-3′ (antisense); hsa-miR-193a inhibitor (anti-miR-193a), 5′-ACUGGGACUUUGUAGGCCAGUU-3′; scrambled miRNA mimic (miR-NC), 5′-UUCUCCGAACGUGUCACGUTT-3′ (sense), 5′-ACGUGACACGUUCGGAGAATT-3′ (antisense); scrambled miRNA inhibitor (anti-NC), 5′-CAGUACUUUUGUGUAGUACAA-3′; EZH2 siRNA (siEZH2), 5′-GCUAAGGCAGCUGUUUCAGTT-3′ (sense), 5′-CU GAAACAGCUGCCUUAGCTT-3′ (antisense); HOTAIR siRNA 1 (si-HOTAIR1), 5′-GCACAGAGCAACUCUAUAATT-3′ (sense), 5′-UUAUAGAGUUGCUCUGUGCTT-3′ (antisense); HOTAIR siRNA 2 (si-HOTAIR2), 5′-GCCUUUGGAAGCUCUUGAATT-3′ (sense), 5′-UUCAAGAGCUUCCAAAGGCTT-3′ (antisense); siRNA negative control (si-NC), 5′-UUCUCCGAACGUGUCACGUTT-3′ (sense), 5′-ACGUGACACGUUCGGAGAATT-3′ (antisense). Human EZH2 cDNA was chemically synthesized and subcloned into Nhel and XhoI restriction site of pcDNA3.1 (+) plasmid (Invitrogen, Carlsbad, CA, USA) to generate pcDNA3.1-EZH2 plasmid which was purchased from Sangon Biotech Co., Ltd. (Shanghai, China). The oligonucleotides and plasmids were transfected using Lipofectamine 2000 (Invitrogen, Carlsbad, CA, USA) following the manufacturer’s protocol. Lentivirus carrying overexpressing Hsa-miR-193a lentiviral vectors (GV309) were from GeneChem (Shanghai, China) and named with LV-miR-193a. LV-shEZH2 is EZH2 knocking down expressing lentivirus, of which double-stranded EZH2-shRNA oligonucleotide was cloned into GV248 vector by GeneChem (Shanghai, China). LV-NC, the GFP vector, was used as control. And the lentiviruses were used to infect cells in the presence of Polybrene. After 48 h, puromycin was added in fresh medium to select of stable clones. Western blot analysis and real-time quantitative reverse transcriptionpolymerase chain reaction (qRT-PCR) were used to determine the efficiency of knockdown and overexpression.

### Data mining and bioinformatics analysis

As previously described [28], we retrieved and re-analyzed the original miRNAs expression and clinical data from the Memorial Sloan Kettering Cancer Center (MSKCC) (www.mskcc.org) and The Cancer Genome Atlas (TCGA) (http://cancergenome.nih.gov) databases to investigate clinical relevance of miR-193a with the pathological traits of patients.

### RNA isolation and real-time quantitative reverse transcription and polymerase chain reaction (qRT-PCR)

Total RNA was extracted from cells or tissues using TRIzol (Takara, Shiga, Japan) according to the manufacturer’s instruction. For miR-193a, qRT-PCR reaction was performed with a HiScript RT kit (VazymeBiotech, Nanjing, China) and AceQ SYBR Green PCR Master Mix (VazymeBiotech) following the manufacturer’s protocols. Primers were chemically synthesized by SprinGen Biotech (Nanjing, China). The miR-193a primers for amplification were as follows: stem–loop reverse transcription primer: 5′-CTCAACTGGTGTCGTGGAGTCGGCAATTCAGTTGAGACTGGGAC -3′; specific forward primer: 5′-ACACTCCAGCTGGGAACTGGCCTACAAAGTCCC -3′; specific reverse primer: 5′-TGGTGTCGTGGAGTCG-3′. The U6 snRNA primers were as follows: forward: 5′-CTCGCTTCGGCAGCACA-3′; reverse: 5′-AACGCTTCACGAATTTGCGT-3′. The HOTAIR primers were: forward: 5′-CAGTGGGGAACTCTGACTCG-3′; reverse: 5′-GTGCCTGGTGCTCTCTTACC-3′; The GAPDH primers were: forward: 5′-TGCACCACCAACTGCTTAGC-3′; reverse: 5′-GGCATGGACTGTGGTCATGAG-3′. PCR was conducted with a 20 μl reaction volume that contained 2 μl of RT products, 1 μl of 10 μM miR-193a or U6 primer set, 10 μl of 2× SYBR Green PCR Master Mix, and 7 μl of ddH2O. Cycling conditions were as follows: 95 °C for 3 min, 95 °C for 15 s, and 60 °C for 60 s. The last two steps were conducted with 40 cycles. qRT-PCR was performed using 7500 Real-Time PCR System (Applied Biosystems, Foster City, CA, USA). The gene expression threshold cycle (CT) values of miR-193a were calculated by normalizing to the internal control U6, and HOTAIR expression was normalized to GAPDH. Relative quantification values were calculated via the 2^−ΔΔCt^ method.

### Cell proliferation assay

PC3 and DU145 cells were seeded at 3 × 10^5^ cells per well in six-well plates and then cultured overnight. At 48 h post-transfection with oligonucleotides or plasmid, cells were trypsinized and seeded at 3000 cells per well in 96-well plates. Cell proliferation was measured by CCK-8 kit (KeyGene Biotech, Nanjing, China) every 24 h according to the manufacturer’s instruction. Absorbance was detected at wavelength of 450 nm. Five wells were measured for cell viability in each treatment group. Three independent experiments were carried out with each 96-well plate.

### Cell colony formation assay

After 48 h transfection, cells were trypsinized and seeded in 6-well plate at a density of 800 cells/well and cultured for 10 to 14 days until colony appeared. Cells were washed with 0.01 M phosphate-buffered saline (PBS, 137 mM NaCl, 10 mM Na2HPO4, 1.8 mM KH2PO4, 2.7 mM KCl, and pH 7.4), fixed with methanol for 20 min, and finally stained with 0.5% crystal violet (make a solution of 0.5% crystal violet in 20% methanol) for 20 min at room temperature. The number of colonies was counted only when they contained more than 50 cells.

### Cell apoptosis assay

Apoptotic cells were stained by using Annexin V-FITC/Propidium Iodide (PI) contained in Apoptosis Kit (KeyGene Biotech, Nanjing, China). After 48 h of transfection, PCa cells were stained with 5 μl Annexin V-FITC and 5 μl PI and cultured in dark at room temperature for 15 min. These cells were analyzed by flow cytometry, and results were processed with Cell Quest ProSoftware (BD Biosciences, San Jose, CA, USA).

In order to label nuclei of apoptotic cells, PC3 and DU145 cells were plated on glass coverslips in 6-well plates, transfected, and fixed in 4% paraformaldehyde 48 h post-transfection. We performed the terminal deoxynucleotidyl transferase-mediated nick-end labeling (TUNEL) staining using an In Situ Cell Death Detection Kit (Roche, Mannheim, Germany) according to the manufacturer’s protocol. The number of TUNEL-positive nuclei was counted and divided by the number of DAPI-stained nuclei to calculate the percentage of TUNEL-positive cells. Three coverslips were analyzed per condition.

### Transwell migration and invasion assay

Migration and invasion assays were performed by using a Transwell chamber with or without Matrigel (BD Biosciences) according to the manufacturer’s instructions. After 48 h of transfection, cells were trypsinized and seeded in top chamber with a density of 1 × 10^5^ cells per well with 200 ul serum-free medium. 600ul of 10% FBS RPMI-1640 medium was added to the lower chamber. Migration and invasion assays were assessed at 8 and 24 h, respectively. Cells on the top chamber were removed, and cells on lower chamber were then fixed with methanol and stained with 0.5% crystal violet and scored.

### In situ hybridization (ISH) and immunohistochemical staining (IHC)

ISH and IHC were performed and subsequent results were analyzed as previously described [29]. In brief, the triple digoxigenin-labeled antisense locked nucleic acid (LNA)-modified probes for HOTAIR and miR-193a were synthesized by Boster Biotech (Wuhan, China). ISH was conducted according to the manufacturer’s instruction of the HOTAIR and microRNA ISH Optimization Kits (Boster, Wuhan, China). IHC was performed using anti-EZH2 antibody (1:250, Abcam, Cambrige, MA, USA) according to the manufacturer’s instructions. The original magnification: ×200.The specific evaluation of gene expression via ISH or IHC was calculated as previously described [30]. In brief, sections with no labeling or less than 5% labeled cells were scored as 0, 5%–30% of cells as 1, 31%–70% of cells as 2, and ≥71% as 3. The staining intensity was scored similarly using 0 for negative staining, 1 for weakly positive, 2 for moderately positive, and 3 for strongly positive. The scores of percentage of positive tumor cells and staining grade were calculated to generate the immune-reactive score for each specimen. A combined score of 0–1 indicates negative expression (−), 2–3 indicates weak expression (+), 4–5 indicated moderate expression (++), and 6 indicates strong expression (+++). Each sample was examined separately and scored by two pathologists [30].

### Xenograft tumor assay

BALB/C nude male mice (six-week old) were purchased from Model Animal Research Center of Nanjing University. All animal experiments were conducted according to the National Institute of Health Guide for the Care and Use of Laboratory Animals and approved by the ethics committee of the Affiliated Zhongda Hospital of Southeast University. PC3 (5 × 10^6^) cells which have been stably infected with LV-miR-193a or LV-NC were subcutaneously injected into each flank of the mice. Tumor size was measured every week with the following formula: (length × width^2^)/2. At the end of experiments, all mice were sacrificed and tumors were dissected and weighed. Partial xenograft tumor tissues were used for RNA extraction and detection of HOTAIR and miR-193a expression. And the rest tissues were formalin fixed immediately and paraffin embedded. Thereafter tissues were stained with antibody against Ki-67, CD31 and CD34 (all 1:100, Boster, Wuhan, China) according to the manufacturer’s instructions.

### RNA sequencing and bioinformatics analysis

PC3 and DU145 cell lines stably overexpressing miR-193a were established via LV-miR-193a infection and puromycin selection. RNA sequencing was assigned to and performed by KangChen Bio-tech Ltd., Co. (Shanghai, China). A total of 2 μg RNA were extracted from LV-miR-193a or LV-NC expressing cells, respectively, and assigned to RNA-sequencing using Illumina-HiSeq4000 system. Gene Enrichment Set Analysis (GSEA) was used to identify gene sets or pathways which were relevant to miR-193a expression profile in PCa cells (http://www.broadinstitute.org/gsea/index.jsp). Hallmark of gene sets were obtained from the Molecular Signatures Database on that website. Normalized enrichment score (NES) and false discovery rate (FDR) were used to analyze across the gene sets.

### Western blot analysis

PC3/DU145 Cells were lysed in RIPA buffer (50 mM Tris-Cl, pH 8.0, 150 mM NaCl, 5 mM EDTA, 0.1% SDS, 1% NP-40) supplemented with 1% proteinase inhibitors (Sigma, St. Louis, MO, USA) and 1 mM PMSF (Beyotime, Hangzhou, China) for total protein preparation. Briefly, 30 μg protein samples were analyzed with 10% SDS-PAGE and then transferred into a polyvinylidene fluoride membrane (Millipore, Billerica, MA, USA). Blots were blocked with 5% skim milk for 1 h at room temperature, and incubated with specific primary antibodies at 4°C overnight. The membranes were washed three times with TBST (TBS-1% Tween 20) buffer and incubated with secondary antibody at room temperature for 1 h, and detected with enhanced chemiluminescence (ECL, Beyotime, Hangzhou, China). Primary antibodies used were rabbit anti-EZH2 (1:1000, Abcam, Cambrige, MA, USA), rabbit anti-H3K27me3 (1:1000, Abcam, Cambrige, MA, USA), rabbit anti-H3(1:1000, Proteintech, Rosemont, IL, USA), HRP-conjugated monoclonal mouse anti-GAPDH (1:1000; KangChen, China;) and HRP-labeled goat anti-rabbit secondary antibody (1:3000; Zhongshan Goldenbridge Biotechnology, Beijing, China).

### Luciferase reporter assay

The exact transcription start site (TSS) of miR-193a has been reported previously [31], 2Kb upstream of TSS were retrieved from NCBI Genome Bioinformatics (http://www.ncbi.nlm.nih.gov/). The promoter region of has-miR-193a was chemically synthesised and cloned into the *Kpn*I and *Hin*dIII restriction sites by GenScript (Nanjing, China), which is downstream the open reading frame of luciferase in the pGL3-basic-Vector (Promega, Madison, WI, USA) to generate the pGL3-miR-193a promoter reporter. For promoter luciferase reporter assay, cells were co-transfected with pGL3-miR-193a promoter and pRL-TK. The 2.3 kb full sequences of wild-type HOTAIR cDNA or mutant HOTAIR cDNA were synthesised by Generay Biotech (Shanghai, China). We then subcloned full cDNA sequence of wild type and mutant HOTAIR into the XhoI and NotI restriction sites of psi-CHECK™-2 vector (Promega, Madison, WI, USA) to generate psiCHECK-HOTAIR-WT and psi-CHECK-HOTAIR-Mut reporters. Luciferase assay activity was measured 48 h after transfection using the Dual-Luciferase Reporter Assay System (Promega, Madison, WI, USA).

### Chromatin immuoprecipitation (ChIP)

PC3 and DU145 cells which were transfected with si-NC or si-HOTAIR2 for 72 h and processed with ChIP assay kit according to the manufacturer’s instructions (Beyotime, China). Antibodies used includes EZH2 antibody (1:50, Abcam, Cambridge, MA, USA), H3K27me3 antibody (1:50, Abcam, Cambridge, MA, USA) and IgG (1:50, Millipore, Billerica, MA, USA). Gene Expression Omnibus (GEO) datasets (DU145 H3K27me3: https://www.ncbi.nlm.nih.gov/geo/query/acc.cgi?acc=GSM1138596; PC3 H3K27me3: https://www.ncbi.nlm.nih.gov/geo/query/acc.cgi?acc=GSM1383872) were retrieved and analyzed for the H3K27me3 peak loci at miR-193a promoter region. ChIP qPCR Primers were specifically designed according to the H3K27me3 loci in PCa cells. The precipitated DNA quantitated by qRT-PCR and normalized by respective 1% ChIP input. ChIP primer sequences used are as follows: sense, 5′-AAAGGCATGATCTGGGTTGGT-3′; antisense, 5′-AGGTCGAGATTTGGAGCCATTTA-3′.

### Statistical analysis

Each value in this study was obtained from at least three independent experiments and presented as mean ± SD. The significance of difference among the means was calculated using Student’s t-tests for two-group comparisons with Statistical Package of the Social Sciences software version 19.0 (SPSS, Inc. Chicago, IL, USA). A two-sided *P* value of <0.05 was considered statistically significant.

## Results

### Experimental scheme and miR-193a expression correlates with prostate cancer clinical features

**Fig. 1:**
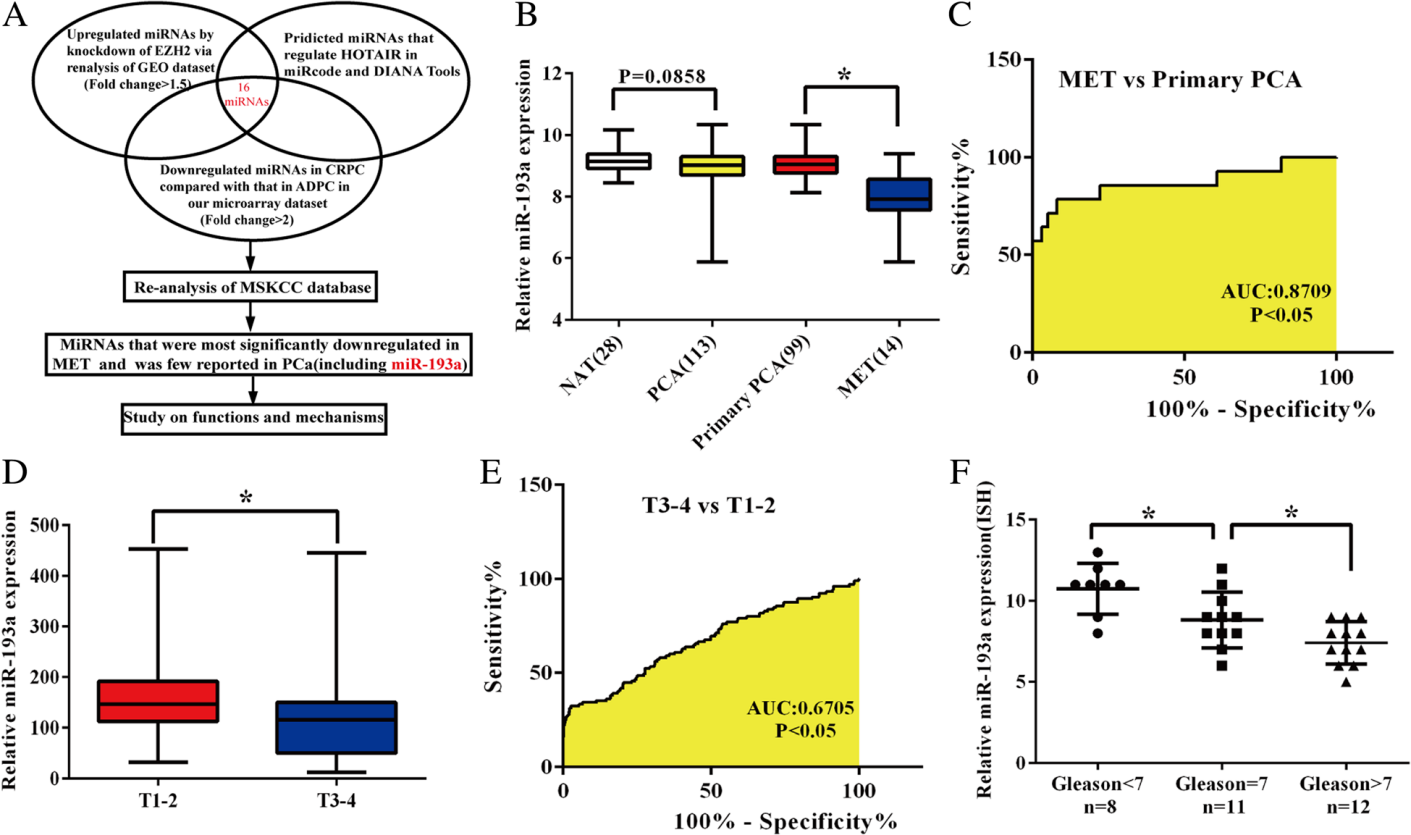
Inverse correlation between miR-193a expression and clinical features in prostate cancer. **a** Experimental scheme. **b** Expression profile of miR-193a in various subgroups of patients from MSKCC database dataset (GSE21032). **c** ROC analysis of MSKCC dataset showing that expression of miR-193a can be used to discriminate metastatic prostate cancer and primary prostate cancer. **d** miR-193a expression by stratifying TCGA dataset. **e** ROC analysis of MSKCC dataset showing the role of miR-193a in discriminating pT1–2 and pT3–4 prostate cancer. **f** Different expression of miR-193a by ISH staining in clinical prostate cancer specimens with different Gleason scores (*n* = 31). *P < 0.05

In our previous microarray analysis [16], we have detected a total of 452 miRNAs that were differentially expressed between CRPC and ADPC (androgen dependent prostate cancer). Among them, 275 miRNAs were significantly downregulated in CRPC with fold change >2, compared with that in ADPC. EZH2 enhances tumorigenesis and is commonly upregulated in various types of cancers including PCa. Its oncogenic activity in cancer mainly depends on EZH2-mediatd epigenetic modification, such as DNA methylation and histone methylation, resulting in aberrantly silencing of tumor suppressive genes in cancer. In order to investigate these potential underexpressed miRNAs which were possibly regulated by epigenetic modification, we reanalyzed the GEO dataset (GSE26996) to identify EZH2 negatively-regulated miRNAs in DU145 [16]. Moreover, to illustrate the potential miRNAs that can regulate HOTAIR in prostate cancer, we searched miRcode algorithm (http://www.mircode.org) and DIANA Tools (http://diana.imis.athena-innovation.gr/DianaTools/index.php?r=lncBase/indexbio). Disciplinary overlaps was showed by intersecting these three groups of miRNAs. 16 miRNAs were shared (Fig. 1a). By aligning these miRNAs to MSKCC prostate cancer dataset (GSE21032) [32], miR-193a was found to be one of the most significantly downregulated miRNAs in metastatic PCa tissues compared with primary cancer (Additional file 1: Table S1). However, few studies have focused on the role of miR-193a in PCa, thus prompting us to focus on it for the present study.

MSKCC dataset includes 28 normal adjacent tissues (NAT), 99 primary prostate cancer tissues (primary PCA), and 14 metastatic prostate cancer tissues (MET). As is shown in Fig. 1b, miR-193a was not significantly underexpressed in primary prostate cancer tissues (*P* = 0.0858). However, its expression in metastatic cancer was significantly decreased compared to primary cancer (*P* < 0.05). ROC analysis demonstrated that the expression of miR-193a can be used to discriminate between metastatic and primary prostate cancers (Fig. 1c). To further validate its expression profile in PCa, the largest TCGA-prostate adenocarcinoma database was investigated and we found that miR-193a expression was significantly downregulated in higher-T stage tumors (Fig. 1d, *P* < 0.05). ROC analysis also showed that the level of miR-193a could be used to discriminate between pT3–4 and pT1–2 PCa (Fig. 1e). All results from MSKCC and TCGA databases imply that miR-193a represents a poor prognostic factor of prostate cancer. To strengthen above findings, we evaluated the miR-193a expression in our clinical specimens via ISH and found that lower level of miR-193a might be correlated with tumor progression as miR-193a was aberrantly downregulated in high-Gleason score tumors (Gleason score: 8–10) (Fig. 1f, *P* < 0.05).

Taken together, all these results suggested that aberrantly expressed miR-193a involves in the progression of prostate cancer and may act as a tumor suppressor.

### Biological effects of EZH2 on PCa cell growth and colony forming in a miR-193a-dependent manner

**Fig. 2:**
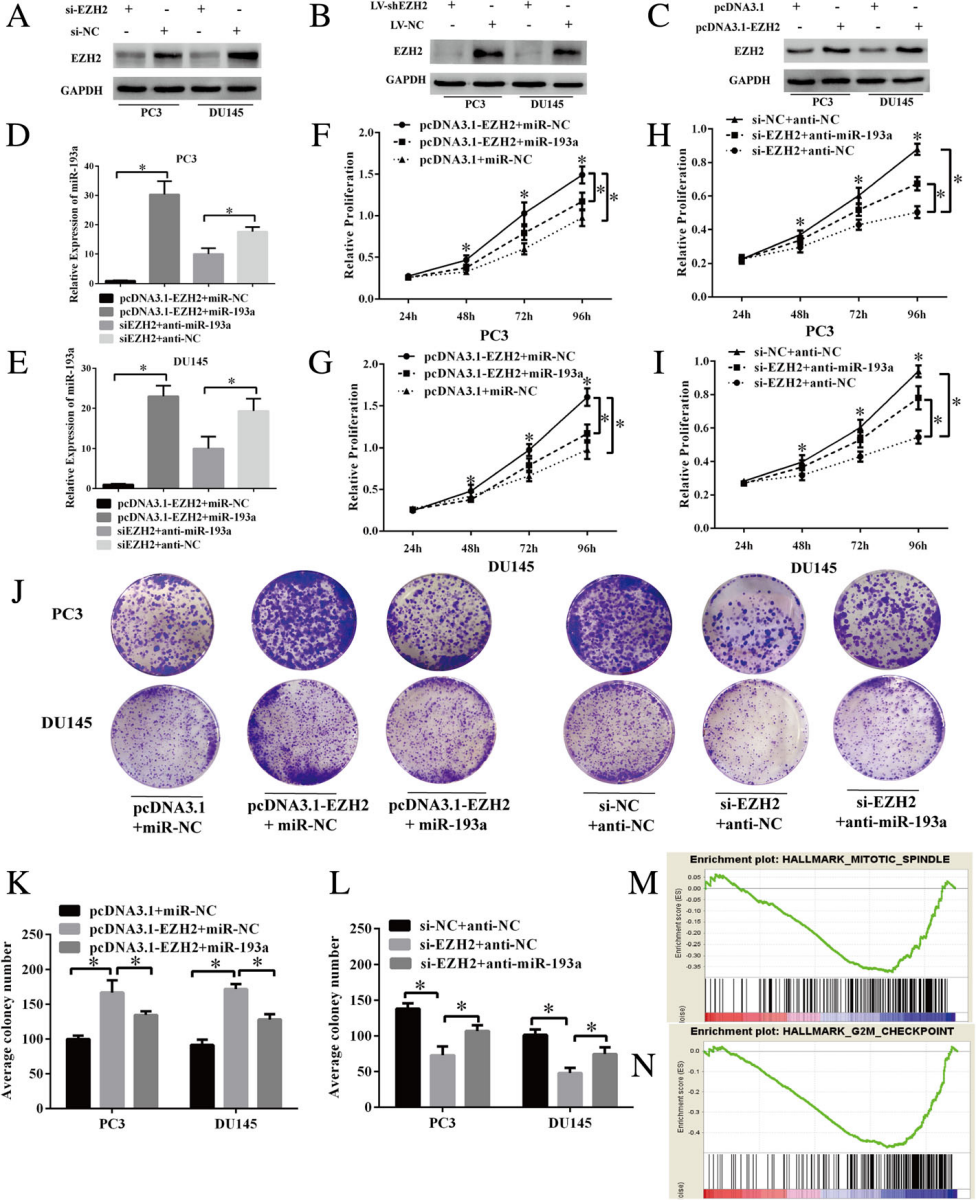
Biological effects of EZH2 on PCa cell growth and colony forming in a miR-193a-dependent manner. **a-c** Western blot assays assessing the knocking down and overexpressing efficiency of si-EZH2, LV-shEZH2 and pcDNA3.1-EZH2 in PC3 and DU145 cell lines, GAPDH was used as an endogenous control. **d–e** The miR-193a expression was measured by qRT-PCR to verify the inhibitory efficiency of si-EZH2 + anti-miR-193a/si-EZH2 + anti-NC and overexpressing efficiency of pcDNA3.1-EZH2 + miR-193a/pcDNA3.1-EZH2 + miR-NC in both cell lines. U6 snRNA was measured as an internal control. CCK8 assays (**f-i**) and colony formation assays (**j-l**) evaluating the biological effects of EZH2 and miR-193a on the cell viability and proliferation in PCa cells. **m-n** GSEA showing that negatively enriched expression of gene sets was involved in hallmarks of mitotic spindle assembly and G2/M cell-cycle checkpoint in miR-193a-overexpressing PCa cells. Each bar represents the mean ± SD of three independent experiments. *P < 0.05

EZH2 was downregulated with si-EZH2 or overexpressed with pcDNA3.1-EZH2 to evaluate its biological effects on cell proliferation in PC3 and DU145 cell lines. The knock-down or overexpression efficiency was verified through western blot analysis (Fig. 2a-c). qRT-PCR was used to assess the inhibitory efficiency for miR-193a after EZH2 depletion and overexpression efficiency for miR-193a after upregulation of EZH2 (Fig. 2d and e). The CCK-8 assay was used to detect the importance of miR-193a in EZH2-associated PCa cell growth and proliferation. Ectopic expression of EZH2 significantly promoted cell viability and proliferation at 48, 72 and 96 h (*P* < 0.05) (Fig. 2f and g). Functional experiments were performed on the EZH2-overexpressing PCa cells supplemented with miR-193a mimics to confirm whether downregulation of miR-193a was required for the EZH2 mediated increase in cell proliferation and viability. Results indicated that miR-193a significantly alleviated the promoting effect of EZH2 on cell growth (Fig. 2f and g). Conversely, anti-miR-193a (miR-193a inhibitor) could also partially abrogate the inhibition effect of proliferation caused by knock-down of EZH2 in PCa cells at 48, 72 and 96 h (*P <* 0.05) (Fig. 2h and i). Similarly, EZH2 overexperssion significantly enhance cell viability of PCa cells based on colony formation assay and miR-193a mimics supplementary partially eliminated the promoting effect. EZH2 depletion significantly inhibited the proliferation of PCa cells while anti-miR-193a alleviated the inhibitory effect (Fig. 2j-l). GSEA showed that a negatively enriched expression of gene sets was involved in mitotic spindle assembly (NES = −1.64, FDR = 0.02, *P* = 0.069) (Fig. 2m) and G2/M cell-cycle checkpoint (NES = −2.0, FDR < 0.05, *P <* 0.05) (Fig. 2n) in miR-193a-overexpressing cells. Collectively, these results indicated that ectopic expression of miR-193a inhibited cell viability and proliferation of PCa cells and EZH2 could modulate cell growth at least in a miR-193a-dependent manner.

### EZH2 modulates apoptosis of PCa cells in a miR-193a-dependent manner and miR-193a suppresses PCa cell migration and invasion in vitro

**Fig. 3:**
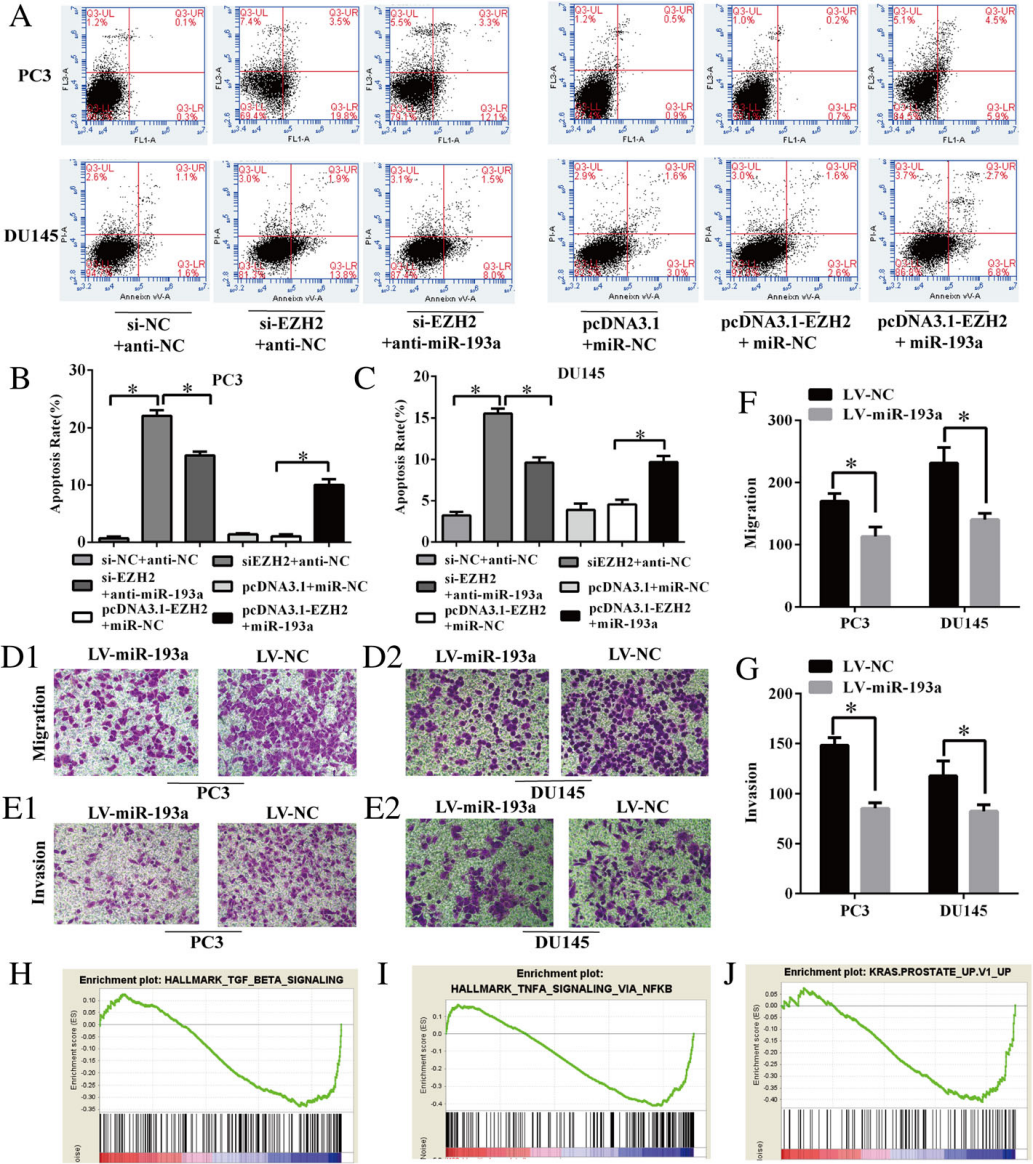
MiR-193a modulates apoptosis and suppresses PCa cell migration and invasion in vitro. (**a**-**c**) Annexin V-FITC/PI apoptosis assays were applied to show the modulation of apoptosis by EZH2 and miR-193a in PC3 and DU145 cells. (**d1**-**d2**, **e1**-**e2**, **f**-**g**) Transwell assays were used to access the influence of overexpressed miR-193a on migration and invasion ability in PC3 and DU145 cells. (**h**-**j**) GSEA showing that negatively enriched expression of gene sets was involved in hallmarks of TGF-β, TNF-α via NF-kB, and KRAS prostate up cell signalings in miR-193a-overexpressing PCa cells. Each bar represents the mean ± SD of three independent experiments. *P < 0.05

EZH2 was downregulated by si-EZH2 and upregulated by transfection with pcDNA3.1-EZH2 to assess the role of EZH2 in PCa cell apoptosis. Meanwhile, the role of miR-193a in PCa cell apoptosis was also explored via transfection with miR-193a mimics after EZH2 depletion or miR-193a inhibitor after overexpression of EZH2. No significant difference was observed between cells transfected with either pcDNA3.1-EZH2 or pcDNA3.1 (*P* > 0.05). EZH2 depletion increased the apoptosis rate in both PC3 and DU145 cells which could be partially rescued by supplementing the cells with anti-miR-193a (apoptosis rate: 22.0 ± 0.6% vs.15.2 ± 0.5% in PC3 and 15.5 ± 0.4% vs. 9.6 ± 0.4% in DU145, *P <* 0.05). On the other hand, upregulation of miR-193a in EZH2-overexpressing cells could significantly induce apoptosis in PCa cells (apoptosis rate: 10.0 ± 0.6% vs.1.1 ± 0.2% in PC3 and 9.7 ± 0.5% vs. 4.6 ± 0.3% in DU145, *P < 0.05*) (Fig. 3a-c). To further elucidate the role of miR-193a on PCa cell apoptosis, we conducted the TUNEL assay (Additional file 2: Figure S3). TUNEL assay also showed similar results as Annexin V-FITC/PI assay did. These results suggested that EZH2 modulated the cell apoptosis of PCa at least partially in a miR-193a-dependent manner.

The aforementioned data linked the downregulation of miR-193a to prostate cancer, especially metastatic cancer, indicating that miR-193a may play a critical role in PCa metastasis. To test this hypothesis, we stably infected human metastatic prostate cancer cell lines (PC3 and DU145) with LV-miR-193a to upregulate the miR-193a expression thereby assessing its impact on cell migration and invasion by performing migration and invasion assays. The results indicated that the overexpression of miR-193a in DU145 and PC3 cell lines strongly suppressed cell migration and invasion abilities (Fig. 3d-g, *P <* 0.05). GSEA showed that a negatively enriched expression of genes sets was involved in hallmarks of transforming growth factor beta (TGF-β) signaling (NES = −1.79, FDR = 0.01, *P* = 0.015) (Fig. 3h), tumor necrosis factor alpha (TNF-α) via nuclear factor kappa-light-chain-enhancer of activated B cells (NF-kB) signaling (NES = −1.77, FDR = 0.005, *P* = 0.017) (Fig. 3i) and KRAS prostate up signaling (NES = −1.69, FDR = 0.08, *P* = 0.203) (Fig. 3j) in miR-193a-overexpressing PCa cells. It is well known that TGF-β signaling plays a significant role in regulation of epithelial mesenchymal transition in PCa by promoting migration and invasion abilities [33]. Moreover, NF-kB signaling could also be activated by TNF-α and enhance the invasion ability of CRPC cells in vitro [34]. Stable knock-down of KRAS signaling has also been reported to suppress PCa cell migration and invasion [35]. In summary, miR-193a could markedly restrain the invasiveness of PC3 and DU145 cells probably by negatively regulating of several pro-invasion signalings.

### Ectopic expression of miR-193a suppresses the formation of prostate xenograft tumors in vivo

**Fig. 4:**
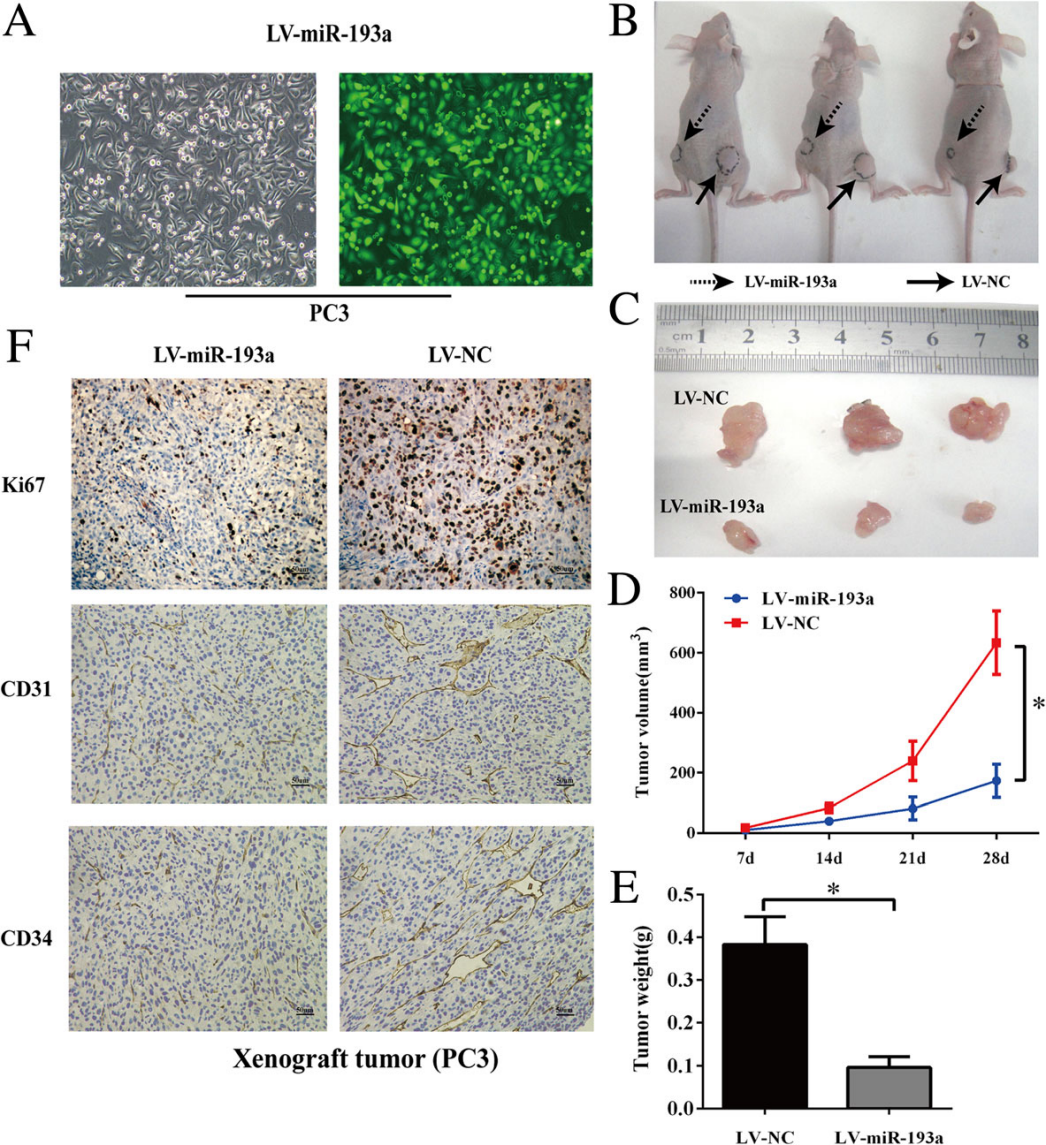
Overexpression of miR-193a suppresses the formation of prostate xenograft tumors in vivo. **a** Fluorescence microscope is used for detecting transfection efficiency for LV-miR-193a transfection. **b** and **c** Subcutaneous tumors formed in nude mice by PC3 cells with stably overexpression of miR-193a or control at 28 days. **d** Tumor volume of miR-193a-overexpressiong PC3 cells at indicated time. **e** Histograms describing the mean tumor weights of each group. **f** Immunohistochemical staining of Ki-67, CD31, and CD34 in the endpoint tumors. Scale bars represent 50 μm. Each bar represents the mean ± SD of three independent experiments. *P < 0.05

To investigate whether miR-193a possesses tumor suppressive ability in vivo. We performed xenograft tumor experiments in nude mice by monitoring tumor incidence, latency and endpoint weight. Stably overexpressing of miR-193a PC3 cells was generated by infecting with lentivirus LV-miR-193a (Fig. 4a) and these cells are then subsequently implanted into nude mice. Results revealed that ectopic expression of miR-193a remarkedly suppressed PCa tumor growth as manifested by reduced tumor size and tumor weight (Fig. 4b-e).

We then carried out immunohistochemical staining of Ki67, CD31 and CD34 in the xenograft tumors (Fig. 4f). The results illustrated that reduced Ki67-positive cells, CD31-positive cells and CD34-positive cells were found in miR-193a-overexpressed xenograft tumor cells. These data suggested that miR-193a suppressed prostate tumor regeneration and growth by inhibition of proliferation, angiogenesis and invasion.

All together, these experiments further confirmed that miR-193a serves as a suppressor in tumorigenesis of prostate cancer.

### EZH2 coupled with HOTAIR to silence miR-193a through introducing trimethylation of H3K27 at miR-193a promoter region

**Fig. 5:**
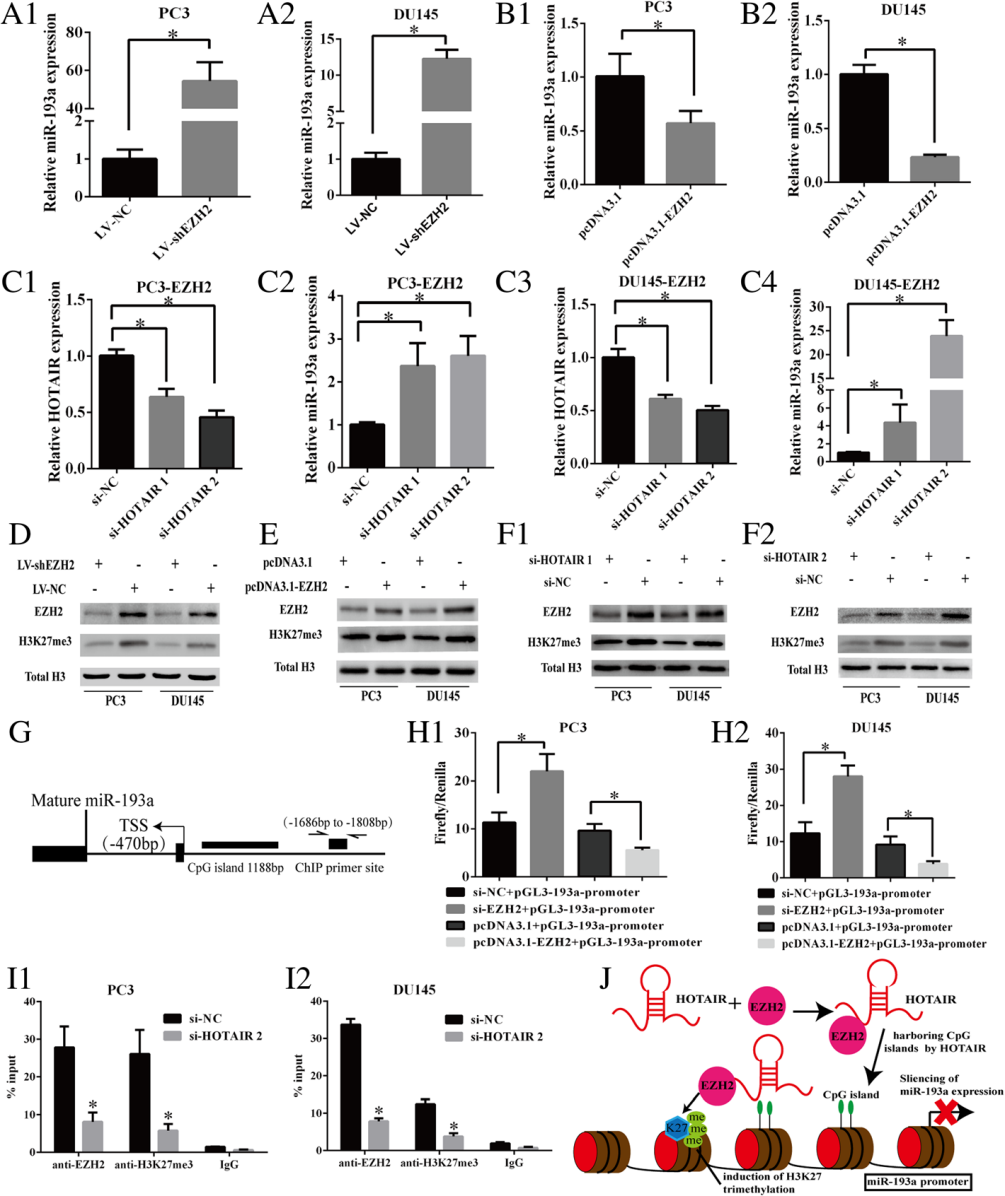
EZH2 coupled with HOTAIR to repress miR-193a expression. (**a1**-**a2**) Expression of miR-193a was measured by qRT-PCR in EZH2 knock-down PC3 and DU145 cells (**b1**-**b2**) or in PC3 and DU145 cell lines with overexpression of EZH2 by transfection of pcDNA3.1-EZH2. U6 snRNA were used as internal controls. (**c1**-**c4**) qRT-PCR assessing the knocking down efficiency of si-HOTAIR and consequent miR-193a expression change in EZH2-overexpressiong PC3 and DU145 cell lines, GAPDH and U6 snRNA were used as an endogenous control, respectively. (**d**-**e**) Western blot assays were performed to measure EZH2 and H3K27me3 expression level in PCa cell lines after depletion of EZH2 by LV-shEZH2 or overexpression of EZH2 by pcDNA3.1-EZH2. Total H3 was used as endogenous control. (**f1**-**f2**) Enrichment of EZH2 and H3K27me3 was measured by western blot in the HOTAIR knock-down PC3 and DU145 cells. (**g**) A diagram of miR-193a gene showing the positions of the mature miR-193a, transcriptional start site (TSS), CpG island and the primer flanking region of the ChIP primer. (**h1**-**h2**) Promoter luciferase reporter assay indicated that depletion of EZH2 by si-EZH2 elevated the promoter activity of miR-193a gene, while upregulation of EZH2 decreased the promoter activity of miR-193a gene in PC3 and DU145 cells. (**i1**-**i2**) ChIP assay showed that knock-down of HOTAIR in PC3 and DU145 cells reduced the enrichment level of EZH2 and H3K27me3 at miR-193a promoter region. (**j**) A hypothetical model illustrating the coupling of EZH2 and HOTAIR to silence miR-193a in PCa cells. Each bar represents the mean ± SD of three independent experiments. *P < 0.05

The in vitro studies have suggested that the oncogenic activities of EZH2 could be contributed by loss of miR-193a in CRPC cell lines. In order to prove that EZH2 had a direct role in repressing miR-193a in PC3 and DU145, we depleted EZH2 by stably infection of lentiviral particles of LV-shEZH2 in PCa cells, and measured the level of miR-193a. Upon inhibition of EZH2, there were upregulation of miR-193a expression in PC3 and DU145 cells (Fig. 5a1-a2, *P <* 0.05). Conversely, we observed that ectopic expression of EZH2 could significantly reduce the level of miR-193a expression (Fig. 5b1-b2, *P <* 0.05) when overexpressing EZH2 by transfecting pcDNA3.1-EZH2 into PC3 and DU145 cells.

It is reported that EZH2 as a component of PRC2 coupled with lncRNA HOTAIR during the gene silencing of tumor suppressor, and the HOTAIR-dependent recruitment of PRC2 was often observed in region enriched with CpG islands [36]. HOTAIR is upregulated in CRPC cell lines and tissues [22, 37], and it is specifically highly expressed in metastatic PCa, we hypothesized that HOTAIR played a role in guiding EZH2 to miR-193a promoter region via recognizing the CpG island (Fig. 5g). To prove that HOTAIR was important in EZH2 mediated silencing of miR-193a, we inhibited the HOTAIR expression by siRNAs in EZH2-overexpressing PCa cells. qRT-PCR indicated that HOTAIR expression was significantly reduced by HOTAIR siRNAs, and inducing higher expression of miR-193a even when EZH2 was overexpressed in PC3 and DU145 cells (Fig. 5c1-c4, *P < 0.05*). These results suggested that EZH2 might couple with HOTAIR to suppress miR-193a in PCa cells.

As we had shown that HOTAIR and EZH2 were critical in repressing miR-193a and silencing of miR-193a contributed to PCa tumorigenesis, we tried to reveal the underlying suppression mechanism of EZH2 on miR-193a. We hypothesized that HOTAIR regulates transcription by directing PRC2 complex via controlling the epigenetic state and downstream gene expression. Western blot assay showed that the enrichment of H3K27me3 significantly decreased when EZH2 was knocked down in LV-shEZH2 infected PCa cells and vice versa (Fig. 5d and e; *P < 0.05*). To test that HOTAIR played a vital role in EZH2-mediated induction of trimethylation of H3K27, we examined expression of EZH2 and H3K27me3 in PCa cells under HOTAIR expression interference. Results showed that both expression of EZH2 and H3K27me3 largely declined in the HOTAIR knocking down PCa cells (Fig. 5f1-f2; *P <* 0.05).

Moreover, we cloned 2.0 kb fragments upstream of TSS of human pri-miR-193a stem-loop into pGL3-basic-vector to generate luciferase construct pGL3-193a-promoter reporter. Then we carried out luciferase reporter assay by co-transfecting pGL3-193a-promoter and si-EZH2 or pcDNA3.1-EZH2 into PC3 and DU145 cells. As expected, co-transfection of the two cell lines with pGL3-193a-promoter and si-EZH2 led to increased luciferase activity significantly. In contrast, co-transfection with pGL3-193a-promoterand pcDNA3.1-EZH2 markedly reduced the luciferase activity in the same cells (Fig. 5h1-h2, *P <* 0.05).These results suggested that EZH2 directly regulates miR-193a activity through its promoter. To address whether HOTAIR regulates miR-193a via EZH2, ChIP assays were conducted to measure the enrichment levels of EZH2 and H3K27me3 at the promoter region of miR-193a. GEO datasets (GSM1383872 and GSM1138596) were reanalyzed to determine the binding site of H3K27me3 at miR-193a promoter region in PC3 and DU145 cells (Fig. 5g). We observed that silencing of HOTAIR led to reduction of EZH2 occupancy and H3K27me3 level at the miR-193a promoter region (Fig. 5i1-i2; *P <* 0.05). It suggests that HOTAIR was required for gene repression effect of EZH2 to miR-193a. Taken together, we showed that EZH2 coupled with HOTAIR to induce H3K27 trimethylation at miR-193a promoter, which reduced miR-193a expression in PC3 and DU145 cells (Fig. 5j).

### Mir-193a directly targets HOTAIR and negatively modulates its expression in PCa

**Fig. 6:**
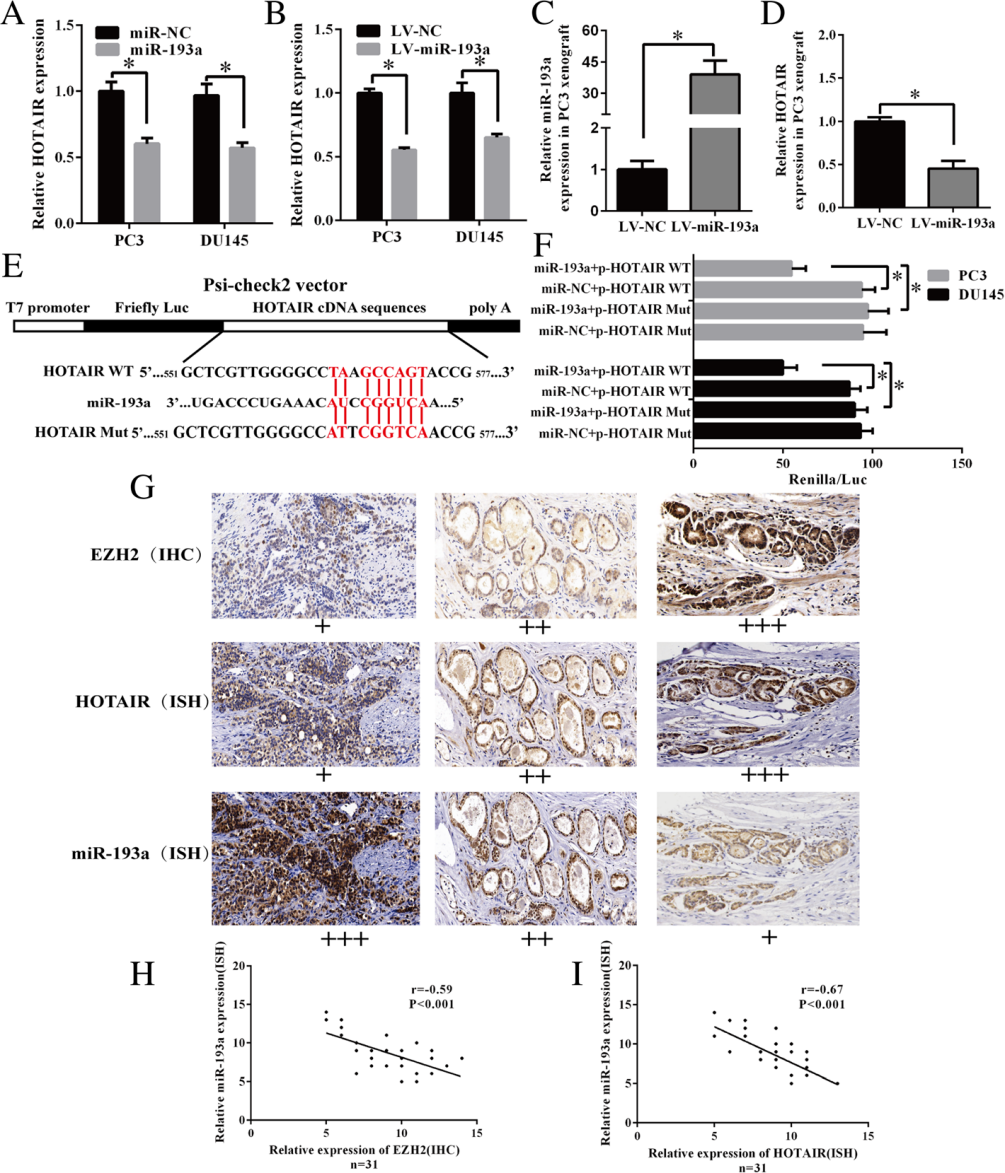
MiR-193a directly targets HOTAIR and negatively modulates its expression in PCa. **a-b** HOTAIR expression was measured by qRT-PCR in PC3 and DU145 cells with overexpression of miR-193a by transfection of miR-193a mimics or infection with LV-miR-193a. GAPDH were used as internal controls. **c-d** Expression of miR-193a and HOTAIR were measured by qRT-PCR in miR-193a-overexpressing PC3 xenograft tumor tissues. U6 snRNA and GAPDH were used as internal controls for miR-193a and HOTAIR, respectively. **e** Schematic showing HOTAIR contained a complementary site to the seed region of miR-193a (551-577 bp) predicted by miRcode and DIANA Tools. **f** The luciferase activity of modified psiCHECK-2 luciferase reporter vector containing wild type or mutations of the binding sites of HOTAIR cDNA. MiR-193a mimics or miR-NC was co-transfected into PC3 and DU145 cells for 48 h. HOTAIR mut as negative control. Rluc activity in the cells was measured and normalized to Fluc activity. **g-i** ISH and IHC staining on 31 PCa clinical tissues with miR-193a/HOTAIR probes and an anti-EZH2 antibody. Spearman correlation analysis showing inverse correlation between HOTAIR and miR-193a as well as between EZH2 and miR-193a. The original magnification: ×200. Each bar represents the mean ± SD of three independent experiments. *P < 0.05

As miRcode algorithm and DIANA Tools showed HOTAIR has miR-193a binding site (Fig. 6e), we wondered whether miR-193a could directly modulate HOTAIR expression. First, we observed ectopic miR-193a expression reduced HOTAIR level in PCa cells either via transfecting with miR-193a mimics or infecting with LV-miR-193a (Fig. 6a and b; *P <* 0.05). Second, HOTAIR expression level was significantly decreased in xenograft tumor tissues which were generated from PC3 cells stably overexpressing miR-193a (Fig. 6c and d; *P <* 0.05). Third, we subcloned full length cDNA sequence of HOTAIR (WT) or HOTAIR (Mut) into psi-CHECK™-2 vector (Fig. 6e), and co-transfected with miR-193a mimics or scramble mimics into PC3 and DU145 cells. The luciferase reporter assay demonstrated that miR-193a significantly reduced luciferase activity (wild type). Mutation of miR-193a binding site in HOTAIR abrogated the inhibitory effects (Fig. 6f, *P <* 0.05). Altogether, above results clearly indicate that miR-193a directly targets HOTAIR and negatively modulates HOTAIR expression in prostate cancer.

Taken all together, we found HOTAIR/EZH2/miR-193a feedback loop in PCa, that HOTAIR is required for EZH2 mediated miR-193a silencing, which in return upregulates HOTAIR expression. In order to further investigate their relationship, we evaluated EZH2, miR-193a and HOTAIR expression through IHC and ISH staining in 31 PCa specimens (Fig. 6g). Spearman correlation analysis showed significant inverse correlation between HOTAIR and miR-193a (*r* = −0.67, *P* < 0.001) as well as between EZH2 and miR-193a (*r* = −0.59, *P* < 0.001) (Fig. 6i). These clinical expression data further supported the HOTAIR/EZH2/miR-193a negative feedback loop.

## Discussion

Here, we identified a HOTAIR-triggered feedback loop that involves EZH2-mediated repression of miR-193a and controls tumorigenesis and prostate cancer progression. This study also provides what we believe is the first in vitro and in vivo proof for a tumor-suppressive function of miR-193a in prostate cancer. We also illustrated a interesting cross-regulation between miR-193a and oncogene HOTAIR that miR-193a directly targets HOTAIR and modulates its expression in prostate cancer. These findings demonstrated the vital role of HOTAIR/EZH2/miR-193a feedback loop in progression of prostate cancer.

The dysregulation of miR-193a has been frequently reported in various types of cancers. Previous studies indicated that miR-193a was markedly downregulated in oral carcinoma, lung cancer, colorectal cancer, and malignant pleural mesothelioma and served as a tumor suppressor in these cancers [9–12]. However, miR-193a also exhibited the promoting effects of chemoresistance in bladder cancer and oesophageal cancer [38, 39]. These findings implied that miR-193a could function either as tumor suppressor or multiple drug resistance gene in a cellular context-dependent manner. Though recently Jia L et al. reported that androgen receptor-regulated miR-193a in C4-2B cells targets AJUBA and promotes migration ability in AR-positive prostate cancer cell lines [40]. However, the expression profile of miR-193a in clinical PCa specimens and the exact biological function of miR-193a in prostate cancer have not been systematically studied. In our current study, we discovered that miR-193a levels were significantly downregulated in metastatic prostate cancer via reanalysis of MSKCC and TCGA datasets, and its expression was negatively correlated with the advanced stages and high gleason scores of PCa patients. Further, from the in vitro and in vivo studies, we showed that miR-193a exhibited its abilities to inhibit cell proliferation, induce apoptosis, suppress migration, invasion and xenograft tumor growth. These results suggested that miR-193a behaved as a tumor suppressor in tumorigenesis of CRPC.

The molecular mechanism that underlie aberrant expression of miRNAs may result from DNA hypermethylation, histone modification, induction of heterochromatin, and alteration of Dicer abundance [41]. It has been reported that miR-193a downregulation is associated with DNA methylation in acute myeloid leukemia, hepatocellular carcinoma, non-small cell lung cancer, and oral cancer [9, 13–15]. To date, little is known about the reason underlying miR-193a silencing in CRPC cells. Cao Q et al. demonstrated that a group of miRNAs was upregulated by EZH2 knock-down in DU145 cell including miR-193a [42], implicating its downregulation in PCa may result from epigenetic modification. Through qRT-PCR and western blot analysis, we showed that H3K27 methyltransferase EZH2 played a vital role in suppression of miR-193a expression in CRPC cells. Luciferase reporter assay revealed that EZH2 could directly bind to the promoter of miR-193a and induce trimethylation of H3K27 thereby silencing miR-193a expression. The interaction between HOTAIR and PRC2 was critical for the regulation of target gene silencing. Tsai et al. demonstrated that PRC2 directly binds to the 5′-end of HOTAIR (1-300 nt). HOTAIR functioned as modular scaffold, guided EZH2 and recruited them to the target gene loci and silence the transcription through trimethylation of H3K27 [36]. Li et al. investigated the binding interactions between PCR2 and HOTAIR, and showed that a loss of miR-34a suppression was observed in HPDE cells expressing HOTAIR lack of EZH2-interacting region, indicating the crucial role of HOTAIR-EZH2 interaction in gene silencing [43]. In this study, we observed that knock-down of HOTAIR led to reduced enrichment of EZH2 and H3K27me3 at the miR-193a promoter region. Interestingly, depletion of HOTAIR could still increase the miR-193a expression even in EZH2-overexpressing PCa cells, implying EZH2-mediated miR-193a suppression was dependent on HOTAIR. Previous study has illustrated that enrichment of the CG-rich motifs in CpG islands was critical for HOTAIR-dependent recruitment of PRC2 in target gene region [36]. A stretch of CpG island could be observed at the promoter region of miR-193a which is important for HOTAIR recognition and subsequently guiding EZH2-associated gene repression. Collectively, EZH2 coupled with HOTAIR to suppress miR-193a expression by epigenetic modification in CRPC cells.

MiRNAs are known as critical modulators of gene expression via post-transcriptional gene silencing. The abundance of numerous lncRNAs could also be regulated by miRNAs [44]. Chiyomaru et al. revealed that miR-141 could directly bind to HOTAIR in a sequence-specific manner and suppress HOTAIR expression, thus inhibiting proliferation and invasion of cancer cells [45]. Tao et al. demonstrated that HOTAIR was a direct target of miR-148 and was inhibited by overexpression of miR-148 in breast cancer [46]. In addition, miR-34a was also shown to lower the stability of HOTAIR in PC3 and DU145 cells [37]. In present study, we found that HOTAIR has the target sequences of miR-193a. Our luciferase reporter assay and real-time PCR results showed that miR-193a binds to the HOTAIR and downregulates HOTAIR expression in PCa cell lines. This study is the first to demonstrate that miR-193a directly targets HOTAIR in both PC3 and DU145 PCa cells.

As miR-193a was epigenetic silenced by EZH2 and HOTAIR, in return the suppression effect of HOTAIR by miR-193a was largely abrogated. Thus, the tumor suppressor miR-193a was continuously inhibited and oncogene HOTAIR remained highly expressed in prostate cancer, which promoting initial prostate cancer developed into highly aggressive cancer type. The clinical expression of these genes in tumor tissues further verified the regulatory mechanism of HOTAIR/EZH2/miR-193a feedback loop in prostate cancer. Therefore, targeting this novel feedback loop would be a promising treatment strategy to prostate cancer.

## Conclusions

In summary, we found that miR-193a is downregulated in metastatic prostate cancer and plays tumor suppressive function in prostate cancer, which was attributed to HOTAIR mediated EZH2 targeting to the promoter of miR-193a. Moreover, HOTAIR is a direct negative target of miR-193a. Thus, a HOTAIR/EZH2/miR-193a feedback loop is formed, which plays a vital role in the progress of CRPC and may be a promising therapy target for PCa treatment.

## Additional files

Additional file 1: Table S1. expression profile of miRNAs with a statistically significant (*P* < 0.05) change in MET and PCA via reanalysis of MSKCC dataset. (PDF 206 kb)

Additional file 2: Figure S3. TUNEL apoptosis assays were applied to show the modulation of apoptosis by EZH2 and miR-193a in PC3 and DU145 cells. (TIFF 1131 kb)

## Abbreviations

ADPC: Androgen dependent prostate cancer
ChIP: Chromatin immuoprecipitation
CRPC: Castration-resistant prostate cancer
EZH2: Enhancer of zeste homolog 2
GEO: Gene Expression Omnibus
GSEA: Gene Set Enrichment Analysis
H3K27me3: Trimethylation of histone H3 lysine 27
HOTAIR: HOX transcript antisense RNA
lncRNA: Long non-coding RNA
MSKCC: Memorial Sloan Kettering Cancer Center
NF-kB: Nuclear factor kappa-light-chain-enhancer of activated B cells
PRC2: Histone methyltransferase polycomb repressor complex 2
TCGA: The Cancer Genome Atlas
TGF-β: Transforming growth factor beta
TNF-α: Tumor necrosis factor alpha
TSS: Transcription start site
