# Supplementary material for: Involvement of aberrantly activated HOTAIR/EZH2/miR-193a feedback loop in progression of prostate cancer
Source: J Exp Clin Cancer Res. 2017 Nov 15;36:159. doi: 10.1186/s13046-017-0629-7 (PMC5688662; doi:10.1186/s13046-017-0629-7)
Supplement: Supplementary file 1 — expression profile of miRNAs with a statistically significant (P < 0.05) change in MET and PCA via reanalysis of MSKCC dataset. (PDF 206 kb) [file 13046_2017_629_MOESM1_ESM.pdf]

Additonal Table S1:MSKCC dataset analysis in MET and PCA tissues

Under-expressed miRNA	Normalized intensity MET	PCA	*P* -value MET/PCA
MiR-193a-3p	7.922 ± 0.273	9.082 ± 0.042	2.72E-05
MiR-148a	11.05 ± 0.411	12.50 ± 0.080	3.65E-05
MiR-203	5.460 ± 0.335	6.556 ± 0.079	8.18E-05
MiR-30c	9.364 ± 0.269	10.41 ± 0.054	9.80E-05
MiR-19b	11.98 ± 0.392	12.59 ± 0.042	1.20E-03
MiR-30b	11.10 ± 0.336	11.70 ± 0.060	3.60E-03
MiR-17	9.762 ± 0.339	10.30 ± 0.053	5.40E-03
MiR-146a	6.709 ± 0.429	7.449 ± 0.101	1.95E-02

MET : metastatic prostate cancer tissues; PCA : primary prostate cancer tissues.
