# Supplementary figures and images for: Involvement of aberrantly activated HOTAIR/EZH2/miR-193a feedback loop in progression of prostate cancer
Source: J Exp Clin Cancer Res. 2017 Nov 15;36:159. doi: 10.1186/s13046-017-0629-7 (PMC5688662; doi:10.1186/s13046-017-0629-7)

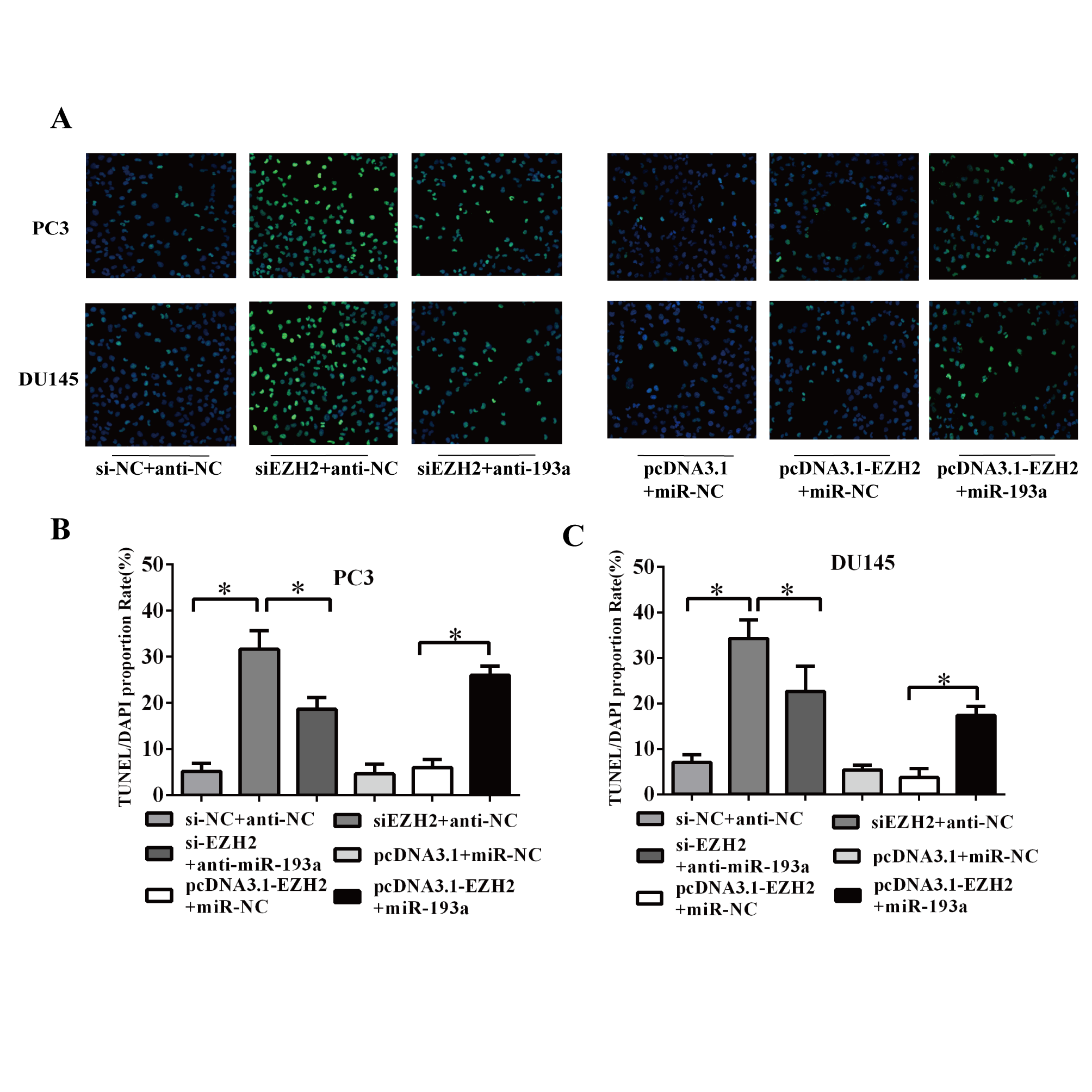

Supplement: Supplementary file 2 — TUNEL apoptosis assays were applied to show the modulation of apoptosis by EZH2 and miR-193a in PC3 and DU145 cells. (TIFF 1131 kb) [file 13046_2017_629_MOESM2_ESM.tif]
